# A rare pancreatic retention cyst mimicking neoplastic lesions: A case report

**DOI:** 10.1016/j.ijscr.2025.111967

**Published:** 2025-09-23

**Authors:** Adriana Ferreira, Filipe Reis Neves, João Correia, Rui C. Oliveira, Rui Miguel Martins

**Affiliations:** a Surgical Department, Instituto Português de Oncologia de Coimbra, Coimbra, Portugal; b Faculty of Medicine, University of Coimbra, Coimbra, Portugal; c Pathology Department, Centro de Diagnóstico Anátomo-Patológico (CEDAP), Coimbra, Portugal; d Center for Investigation on Environment, Genetics and Oncobiology (CIMAGO), Faculty of Medicine, University of Coimbra, Coimbra, Portugal

**Keywords:** Pancreatic cysts, Retention cyst, IPMN, Differential diagnosis, Case report

## Abstract

**Introduction:**

Pancreatic cysts are increasingly detected due to the widespread use of high-resolution imaging. These cysts range from benign to malignant and often require careful evaluation to guide management. Differentiating between neoplastic and non-neoplastic cysts remains a diagnostic challenge. Among the non-neoplastic types, pancreatic retention cysts are rare and often under-recognized.

**Case presentation:**

We present the case of an asymptomatic woman in her 50s referred to our oncology center for further investigation of a pancreatic cystic lesion initially discovered incidentally. Imaging follow-up revealed a growth of 2.5 mm in one year. MRI, CT, and endoscopic ultrasound findings, combined with fine-needle aspiration cytology, suggested a mucinous neoplasm—most likely an intraductal papillary mucinous neoplasm (IPMN). The patient underwent distal pancreatectomy with splenectomy. Histopathological analysis revealed a pancreatic retention cyst with no evidence of malignancy.

**Clinical discussion:**

Retention cysts, though benign, may mimic the radiological and cytological features of IPMNs, leading to overtreatment. This case highlights the importance of integrating clinical, imaging, and histopathological data to reach an accurate diagnosis. The limitations of cytology in cystic lesions and the potential value of molecular testing should be considered in equivocal cases.

**Conclusion:**

This case underscores the diagnostic complexity of pancreatic cystic lesions. It emphasizes the need for cautious interpretation of fine-needle biopsy findings and highlights the potential diagnostic bias introduced by fragmented care.

## Highlights

• Pancreatic cysts have a wide differential diagnosis, ranging from benign to malignant entities.
• This case report describes a rare retention cyst initially misdiagnosed as an IPMN.
• It emphasizes the importance of integrating clinical, radiological, and pathological findings.
• Retention cysts, though benign, can mimic malignant lesions and lead to overtreatment.
• Proper interpretation of biopsy and imaging findings can prevent unnecessary surgery.

## 1 Introduction

The prevalence of pancreatic cysts is estimated to range from 0.2 % to 44.7 % [[1], [2], [3], [4], [5]]. This significant variation can be partially attributed to the different imaging methods employed for diagnosis, as varying incidences of pancreatic cysts have been reported depending on whether Magnetic Resonance Imaging (MRI), Computed Tomography (CT), or a combination of several exams [6]. Pancreatic cysts can be categorized into two main groups: neoplastic and non-neoplastic cysts. In the neoplastic category, cysts can be further classified into serous cysts, which are typically regarded as benign lesions, and mucinous cysts. Mucinous cysts are associated with malignant potential and encompass intraductal papillary mucinous neoplasms (IPMNs) and mucinous cystic neoplasms (MCNs). In contrast, non-neoplastic cysts are benign lesions that include a variety of types, such as pseudocysts, retention cysts, and epithelial cysts [7]. Besides being classified as neoplastic or non-neoplastic, pancreatic cysts can also be subdivided into epithelial and non-epithelial categories according to WHO criteria [5].

The advancements in imaging technologies and the widespread adoption of high-resolution imaging have resulted in a greater number of identified pancreatic neoplasms. Despite these technological improvements, it remains challenging in some cases to differentiate between non-neoplastic and neoplastic pancreatic cysts [8]. In accordance with the aforementioned, it is essential to accurately diagnose and treat this pathology, given that its biological behaviour can range from benign to malignant disease.

This case report illustrates an instance of a pancreatic retention cyst that was presented and managed as an IPMN. More specifically, in this case, we observed an initial diagnostic investigation that suggested the presence of a mucinous neoplasia. This finding consequently led to a therapeutic approach as if it were an IPMN. However, after surgical treatment, the definitive histopathological diagnosis revealed a retention cyst, which requires a different therapeutic orientation. In terms of clinical practice, this case serves as an excellent example of how the differential diagnosis of pancreatic cysts can be challenging and highlights the necessity of remaining vigilant for the existence of less common entities.

This case report has been reported in line with the SCARE checklist [9].

## 2 Case presentation

A woman in her 50s was referred to our oncology hospital for evaluation and follow-up of a pancreatic lesion, which was initially diagnosed incidentally during an ultrasound requested by her general practitioner.

The patient had a history of hypertension, dyslipidemia, and depression, and denied any previous history of acute pancreatitis. She reported no symptoms and denied excessive smoking or alcohol consumption. The general examination was unremarkable.

The pancreatic cyst had first been diagnosed and monitored at another hospital abroad. Only the reports of previous imaging (CT and MRI) were available, without direct access to the images themselves. These reports documented the size of the lesion, which, when compared with the new imaging performed at our facility, confirmed a cystic growth of 2.5 mm over the course of one year.

## 3 Investigations

**Fig. 1:**
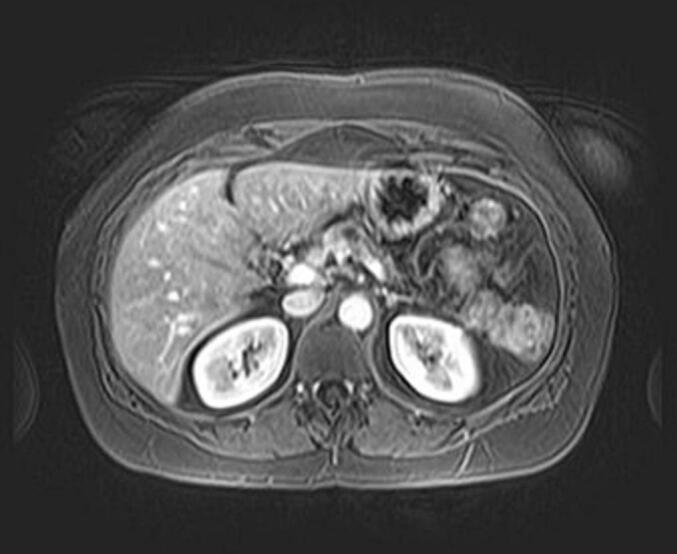
Magnetic resonance imaging (MRI) showing a cystic lesion in the body of the pancreas, in close contact with the splenic vessels.

**Fig. 2:**
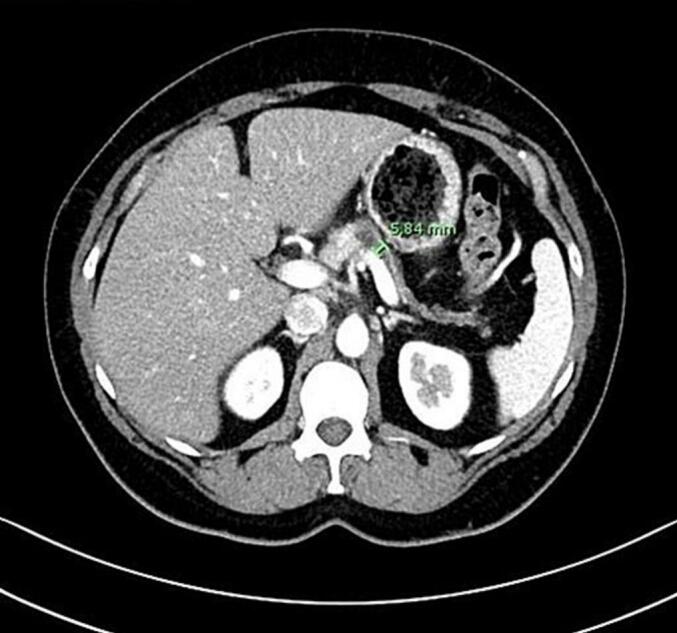
Axial contrast-enhanced computed tomography (CT) scan showing the main pancreatic duct measuring 5.84 mm.

Serum carcinoembryonic antigen (CEA) and carbohydrate antigen 19.9 (CA 19.9) tests returned normal results, with CEA measuring 2.20 ng/mL and CA 19.9 at 29.5 U/mL. Serum amylase levels were slightly elevated at 202 U/L. MRI and CT scans revealed a cystic lesion measuring 28 mm in the body of the pancreas, characterized by T1 hypointensity and T2 hyperintensity, along with an intermediate signal area suggestive of a cyst. The lesion appeared to be in close proximity to the pancreatic duct, which measured 5.84 mm. This lesion might correspond to an intraductal papillary mucinous neoplasm (IPMN), and the possibility of malignant degeneration cannot be excluded due to the observed enhancement (Fig. 1, Fig. 2).

Ultrasound endoscopy revealed a hypoechoic, heterogeneous area with poorly defined contours and calcifications located in the body of the pancreas, measuring approximately 28.0 × 11.2 mm in diameter in the sagittal plane. A fine-needle biopsy was performed, which was consistent with a pancreatic mucinous neoplasm without any obvious criteria for malignancy.

## 4 Differential diagnosis

Based on the imaging and biopsy results, the primary differential diagnosis was between a main-duct IPMN and a mucinous cystic neoplasm.

## 5 Treatment

**Fig. 3:**
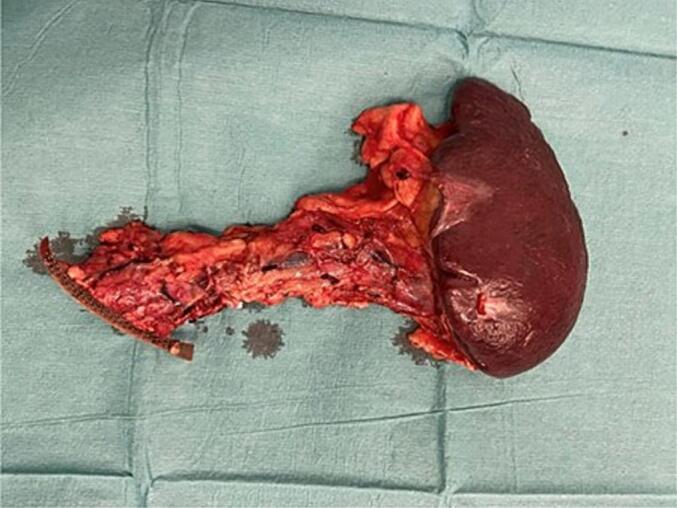
Distal pancreatectomy with en-bloc splenectomy.

The case was discussed in a multidisciplinary tumor board meeting and, integrating the imaging findings and the biopsy results, surgical intervention was proposed. Intraoperatively, a pancreatic lesion with poorly defined edges was identified using ultrasound. Due to its close proximity to the splenic artery and the potential for malignant degeneration associated with the lesion, we decided to perform a distal pancreatectomy with en-bloc splenectomy (Fig. 3).

## 6 Anatomopathological diagnosis

**Fig. 4:**
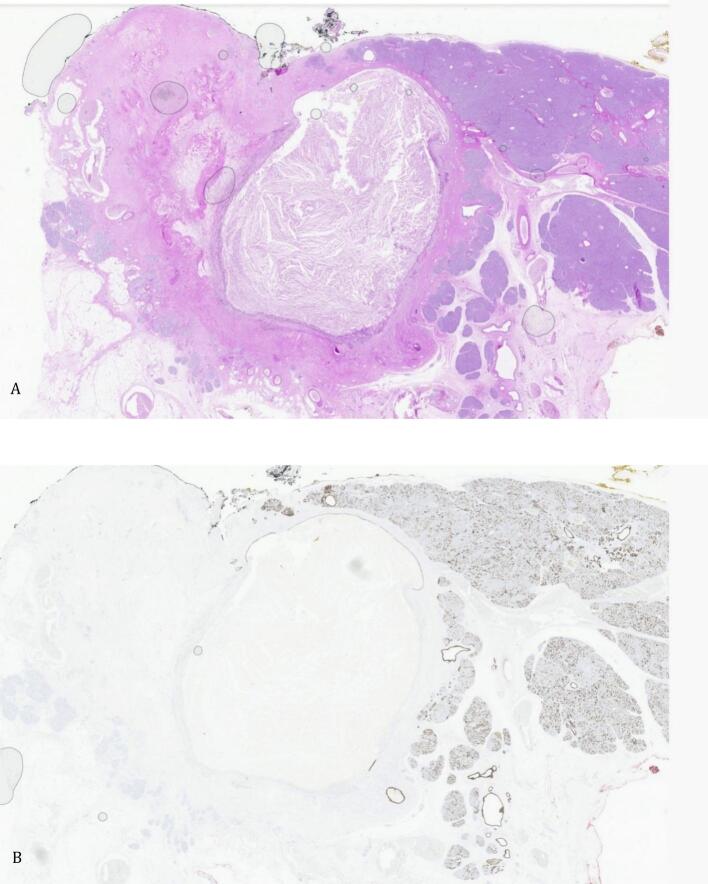
Pathological examination revealed the diagnosis of a retention cyst measuring 2.6 cm, characterized by ulceration of the lining, without evidence of malignancy or mucus production, and accompanied by localized chronic pancreatitis. A review of the cytology specimen exposed the presence of fibrin and foamy macrophages, with occasional mucus; however, no cylindrical epithelium was observed. A. 20× H&E; B. 20× CK7.

Pathological examination revealed the diagnosis of a retention cyst measuring 2.8 cm, characterized by ulceration of the lining, without evidence of malignancy or mucus production, and associated with localized chronic pancreatitis (Fig. 4A and B).

## 7 Outcome and follow-up

The postoperative period was uneventful, and the patient was discharged on the fourth postoperative day. Given the benign nature of the lesion, no follow-up is necessary.

## 8 Discussion

We present a case of an asymptomatic patient with an incidental pancreatic cyst.

In fact, only one-third of patients with IPMN are symptomatic, often presenting with epigastric or back pain, weight loss, acute pancreatitis, jaundice, or new-onset diabetes. Similarly, patients with mucinous cysts may exhibit nonspecific symptoms; however, it is more common for these patients to be completely asymptomatic [10].

The literature on pancreatic retention cysts primarily consists of articles that describe various rare pancreatic cysts. Consequently, research was conducted on the PubMed platform to identify articles that specifically address rare pancreatic cysts, including retention cysts. Additionally, regarding the literature on the management of pancreatic cysts, particularly the approach to IPMNs, the research was largely based on the following sources: the European Evidence-Based Guidelines on Pancreatic Cystic Neoplasms (2018), the International Evidence-Based Kyoto Guidelines for the Management of Intraductal Papillary Mucinous Neoplasm of the Pancreas (2023), the ACG Clinical Guideline for the Diagnosis and Management of Pancreatic Cysts (2018), and the American Gastroenterological Association Institute Guideline on the Diagnosis and Management of Asymptomatic Neoplastic Pancreatic Cysts (2015).

Due to the growth of cysts, an investigation was conducted, which included serum CEA and CA 19.9 tests, MRI, endoscopic ultrasound, and fine-needle biopsy. Specifically, CA 19.9 should be considered in IPMN when there is concern for malignant transformation. The accuracy of MRI/magnetic resonance cholangiopancreatography (MRCP) for identifying the specific type of pancreatic cyst ranges from 40 % to 95 %, while for CT, it ranges from 40 % to 81 %. Endoscopic ultrasound (EUS) is recommended as an adjunct to other exams, with the contrast-enhanced harmonic modality being considered for further evaluation of mural nodules [5,11]. Endoscopic ultrasound fine-needle aspiration (EUS-FNA) enhances diagnostic accuracy in pancreatic cysts and should be performed whenever the results could influence the management of the condition. However, it demonstrates good specificity but poor sensitivity in differentiating benign from malignant IPMNs [12]. Furthermore, recent studies have suggested that preoperative next-generation sequencing (NGS) of pancreatic cyst fluid (PCF) for KRAS and GNAS mutations is highly sensitive for IPMNs and specific for mucinous pancreatic cysts, making it possible to utilize the analysis of these mutations in cases where the diagnosis is unclear [5,13].

A cystic growth of 2.5 mm within a year was the characteristic that prompted further investigation of the lesion. Several features are recognized as risk factors for the malignant progression of an IPMN, such as the presence of jaundice, an enhancing mural nodule with a diameter of 5 mm or more, a solid component, positive cytology, or a main pancreatic duct measuring 10 mm or more. Additional factors associated with an increased risk of high-grade dysplasia or cancer include a main pancreatic duct measuring between 5 and 9.9 mm, a cystic growth rate of at least 2.5 mm per year, elevated serum levels of CA 19.9, the presence of symptoms, enhancing mural nodules of any size, and a cyst diameter of at least 40 mm [5,6]. It is important to note that the progression of IPMNs that do not meet surgical criteria to lesions requiring resection can occur at any time following diagnosis, underscoring the necessity for lifelong follow-up of these patients. Considering all these factors, it is crucial to balance the risk of cancer and the need for surgery with the requirement for long-term follow-up in younger individuals [5,14].

In this case, following the biopsy results indicating a pancreatic mucinous neoplasm and after careful discussion with the patient, a distal pancreatectomy with en-bloc splenectomy was proposed and successfully performed without complications. While several features — including mild main pancreatic duct dilation, the presence of calcifications, and the absence of definite mural nodules — could have favored a benign lesion such as a retention cyst, both a main duct diameter of 5.84 mm (within the 5–10 mm risk range) and a documented cyst growth of 2.5 mm over one year are recognized risk factors for high-grade dysplasia or neoplasia in the setting of suspected IPMN. Moreover, radiological reports explicitly mentioned the possibility of an IPMN, and the lesion's proximity to the splenic artery further reinforced the malignant potential considered at the time. Taken together, these elements supported the surgical intervention performed as a reasonable, though debatable, option in this clinical context. Vaccination against encapsulated organisms (*Streptococcus pneumoniae*, *Haemophilus influenzae* type b, and *Neisseria meningitidis*) was administered two weeks prior to the procedure, in accordance with recommendations [15]. In the literature, there remains a lack of consensus regarding the surgical treatment strategies for main duct/mixed type IPMN [16]. Some surgeons advocate for total pancreatectomy in every patient with radiological involvement of the entire main pancreatic duct, while others recommend total pancreatectomy only in cases with a family history of pancreatic cancer, or suggest a partial pancreatectomy followed by close surveillance, with total pancreatectomy performed if progression or recurrence is suspected [5]. The use of frozen section analysis during partial pancreatectomy has been validated, as it allows the surgeon to determine whether to extend the resection based on the results [5,16]. An organ-preserving pancreatectomy may be considered if a non-invasive lesion is suspected, as this approach can reduce morbidity due to its less invasive nature and the preservation of the pancreas's endocrine and exocrine functions [6,17,18]. Spleen-preserving pancreatectomy is also a viable option [19]. Given the established advantages of minimally invasive techniques, laparoscopic or robotic pancreatectomy should also be considered [6].

The pathological examination revealed a diagnosis of a retention cyst. Recognizing the discordance, the pathology department reviewed the cytology and reported: “A review of the cytology specimen showed fibrin and foamy macrophages, with occasional mucus, but no cylindrical epithelium was evident. With the clinical suspicion of IPMN, the sample was rendered as compatible with a mucinous neoplasm.”

The initial diagnosis of IPMN in cytology should be approached with caution. While distinctions between IPMN and retention cysts are clear histologically, cytological evaluation of pancreatic cysts has important limitations. A retention cyst may contain retained mucin, but the absence of epithelial cells warrants careful consideration. EUS-FNA cytology is a valuable initial approach; however, it has certain limitations and should always be correlated with clinical and imaging findings to avoid potential misdiagnosis. In fact, cytology often cannot reliably differentiate between IPMN, mucinous cystic neoplasms, and benign cysts with mucin, particularly in small or scant samples. Moreover, contamination from normal duodenal mucus during endoscopic ultrasound-guided aspiration has been shown to mimic IPMN cytology [20]. Additionally, analyzing genetic features in mucus or cytology samples may enhance diagnostic accuracy, as IPMNs typically exhibit GNAS mutations, whereas retention cysts do not [21,22].

Retention cysts are cystic dilations of the pancreatic duct caused by intraluminal obstruction, which can be congenital or secondary to factors such as calculi, chronic pancreatitis, mucin accumulation, or pancreatic adenocarcinoma [7]. They are typically small lesions, and their walls are lined by nonciliated simple cuboidal or columnar epithelium, often reflecting attenuation of the native duct lining, and may contain mucin. They characteristically communicate with the pancreatic duct. In contrast to IPMN, they lack mural nodules or a solid component and typically do not cause downstream ductal dilation [23,24]. This condition is rare, with a prevalence ranging from 0.1 % to 1.2 % in series of resected cystic lesions of the pancreas. It is more commonly observed in patients with cystic fibrosis, appearing in approximately 25 % of these individuals [25,26].

Additionally, it is important to note that pancreatic cysts, including retention cysts, have been found in up to 15 % of patients with pancreatic cancer [26].

Patients with surgically resected benign cysts, including retention cysts, do not require any follow-up after resection [27,28]; therefore, the patient was discharged from the surgery follow-up appointments.

IPMN are cystic neoplasms of the pancreas that develop from the epithelial cells lining the pancreatic ducts, with the majority found in the head of the pancreas. It is a mucin-producing neoplasm of the exocrine pancreas, characterized by intraductal papillary projections lined by mucinous epithelium. They frequently demonstrate production of thick mucin and are associated with dilatation of the main pancreatic duct or its branches [24]. These neoplasms are more commonly diagnosed in individuals during the seventh and eighth decades of life [29,30]. IPMNs have the potential to become malignant, and the associated risk varies depending on the specific type. IPMNs arising from the accessory duct have a lower risk of malignancy (2–25 %) compared to those originating from the main duct and mixed types (33–60 %) [31].

As observed in this case and supported by literature and other reported instances, it can be challenging to establish a differential diagnosis between these lesions. A comprehensive evaluation — integrating clinical history, risk factors, radiological features, cytology, and molecular data — is crucial in guiding management. In young patients, it is particularly important to balance the risk of overtreatment against the risk of indefinite surveillance.

This case further illustrates the challenges associated with fragmented care. The patient had been followed in different institutions and countries, which limited access to prior examinations and a comprehensive medical history, and the absence of a unified diagnostic pathway likely contributed to the decision for surgical intervention. While distal pancreatectomy with splenectomy is a major procedure with potential morbidity, the presumed malignant potential strongly influenced the decision to proceed. Given her young age, indefinite surveillance would otherwise have been required, involving repeated imaging (including CT scans with cumulative radiation exposure) and the psychological burden of living with the possibility of harboring a premalignant lesion.

Ultimately, this case underscores the importance of maintaining a broad differential diagnosis, including benign entities such as retention cyst or post-inflammatory pseudocyst, and avoiding anchoring bias. It also highlights the need for a truly multidisciplinary approach and structured diagnostic pathways to minimize overtreatment in pancreatic cystic lesions. Discussing the risks and benefits of each management strategy with the patient is considered a best practice in clinical care for nearly all pathologies, and it is particularly crucial in the management of pancreatic cysts.

## 9 Conclusion

Retention cysts of the pancreas are rare and may mimic neoplastic lesions both clinically and radiologically. This case emphasizes the need for cautious interpretation of fine-needle biopsy findings and highlights the potential diagnostic bias introduced by fragmented care. Multidisciplinary discussion and histopathological analysis remain essential to ensure accurate diagnosis and minimize unnecessary surgical intervention.

## CRediT authorship contribution statement

Adriana Ferreira: Conceptualization, data collection, writing—original draft, surgical management of the patient.
Filipe Reis Neves: Data interpretation, writing—review and editing.
João Correia: Patient management, review of literature.
Rui C Oliveira: Histopathological analysis, figure preparation, critical revision.
Rui Miguel Martins: Supervision, final manuscript approval, surgical management and guidance.

## Consent

Written informed consent was obtained from the patient for publication of this case report and accompanying images. A copy of the written consent is available for review by the Editor-in-Chief of this journal on request.

## Ethical approval

Ethical approval was not required for this case report in accordance with institutional policies.

## Guarantor

Adriana Ferreira; Rui Miguel Martins.

## Research registration number

Not applicable.

## Funding

This research did not receive any specific grant from funding agencies in the public, commercial, or not-for-profit sectors.

## Declaration of competing interest

The authors declare that they have no known competing financial interests or personal relationships that could have appeared to influence the work reported in this paper.

## References

[1] Majumder S., Chari S.T. (Aug 4 2023).

[2] Chang Y.R., Park J.K., Jang J.Y. (Dec 2016). Incidental pancreatic cystic neoplasms in an asymptomatic healthy population of 21,745 individuals. Medicine.

[3] de Jong Koen, Bruno M.J., Fockens P. (Jan 1 2012). Epidemiology, diagnosis, and management of cystic lesions of the pancreas. Gastroenterol. Res. Pract..

[4] Kromrey M.L., Bülow R., Hübner J. (Sep 6 2017). Prospective study on the incidence, prevalence and 5-year pancreatic-related mortality of pancreatic cysts in a population-based study. https://gut.bmj.com/content/gutjnl/67/1/138.full.pdf.

[5] (Mar 24 2018). Gut [Internet].

[6] Ohtsuka T., Fernández-del Castillo C., Itoi T. (2023). International evidence-based Kyoto guidelines for the management of intraductal papillary mucinous neoplasm of the pancreas. Pancreatology.

[7] Kim Y.S., Cho J.H. (2015). Rare nonneoplastic cysts of pancreas. Clin. Endosc..

[8] de Jong Koen, Yung Nio C., Hermans J.J. (Sep 1 2010). High prevalence of pancreatic cysts detected by screening magnetic resonance imaging examinations. Clin. Gastroenterol. Hepatol..

[9] Kerwan A., Al-Jabir A., Mathew G., Sohrabi C., Rashid R., Franchi T., Nicola M., Agha M., Agha R.A. (2025). Revised Surgical CAse REport (SCARE) guideline: an update for the age of Artificial Intelligence. Premier J. Sci..

[10] Singh R.R., Gopakumar H., Sharma N.R. (Feb 2 2023). Diagnosis and management of pancreatic cysts: a comprehensive review of the literature. Diagnostics.

[11] Fusaroli P., Serrani M., Lisotti A. (2017). Role of contrast harmonic-endoscopic ultrasound in pancreatic cystic lesions. Endosc. Ultrasound.

[12] Suzuki R., Thosani N., Annangi S., Guha S., Bhutani M.S. (Sep-Oct 2014). Diagnostic yield of EUS-FNA-based cytology distinguishing malignant and benign IPMNs: a systematic review and meta-analysis. Pancreatology.

[13] Singhi A.D., McGrath K., Brand R.E. (2018). Preoperative next-generation sequencing of pancreatic cyst fluid is highly accurate in cyst classification and detection of advanced neoplasia. Gut.

[14] Del Chiaro Marco, Ateeb Zeeshan, Hansson Marcus Reuterwall (Apr 1 2017). Survival analysis and risk for progression of intraductal papillary mucinous neoplasia of the pancreas (IPMN) under surveillance: a single-institution experience. Ann. Surg. Oncol..

[15] Bonanni P., Grazzini M., Niccolai G. (Dec 8 2016). Recommended vaccinations for asplenic and hyposplenic adult patients. Hum. Vaccin. Immunother..

[16] Scholten L.L., van Huijgevoort N.C., Bruno M.M. (2019). Surgical management of main duct IPMN and mixed type IPMN: an international survey and case-vignette study among experts. HPB.

[17] Beger H.G., Mayer B., Poch B. (Nov 1 2023). Duodenum-preserving pancreatic head resection for benign and premalignant tumors—a systematic review and meta-analysis of surgery-associated morbidity. J. Gastrointest. Surg..

[18] Pausch T.M., Liu X., Dincher J. (Mar 3 2023). Middle segment-preserving pancreatectomy to avoid pancreatic insufficiency: individual patient data analysis of all published cases from 2003–2021. J. Clin. Med..

[19] Kimura W., Moriya T., Ma J. (2007). Spleen-preserving distal pancreatectomy with conservation of the splenic artery and vein. World J. Gastroenterol..

[20] Stelow E.B., Stanley M.W., Bardales R.H. (Sep 2003). Intraductal papillary-mucinous neoplasm of the pancreas. The findings and limitations of cytologic samples obtained by endoscopic ultrasound-guided fine-needle aspiration. Am. J. Clin. Pathol..

[21] Abdelkader A.A., Hunt B.B., Hartley C.C.P. (2020). Cystic lesions of the pancreas: differential diagnosis and cytologic–histologic correlation. Arch. Pathol. Lab. Med..

[22] Takano S.S., Fukasawa M.M., Maekawa S.S. (2014). Deep sequencing of cancer-related genes revealed GNAS mutations to be associated with intraductal papillary mucinous neoplasms and its main pancreatic duct dilation. PLOS ONE.

[23] Karoumpalis I., Christodoulou D.K. (2016). Cystic lesions of the pancreas. Ann. Gastroenterol..

[24] Abdelkader A., Hunt B., Hartley C.P. (2020). Cystic lesions of the pancreas: differential diagnosis and cytologic-histologic correlation. Arch. Pathol. Lab Med..

[25] Esposito I., Häberle L. (Jan 1 2022). Encyclopedia of Pathology.

[26] Itai Yuji, Moss A.A., Goldberg H.I. (Aug 1 1982). Pancreatic cysts caused by carcinoma of the pancreas. J. Comput. Assist. Tomogr..

[27] Elta G.H., Enestvedt B.K., Sauer B.G. (Apr 2018). ACG clinical guideline: diagnosis and management of pancreatic cysts. Am. J. Gastroenterol..

[28] Vege S.S., Ziring B., Jain R. (Apr 2015). American Gastroenterological Association Institute guideline on the diagnosis and management of asymptomatic neoplastic pancreatic cysts. Gastroenterology.

[29] Volkan Adsay N. (Feb 2007). Cystic lesions of the pancreas. Mod. Pathol..

[30] Puckett Y., Sharma B., Kasi A. (2020). https://www.ncbi.nlm.nih.gov/books/NBK507779/.

[31] Babiker H.M., Hoilat G.J., Menon G. (2021). https://www.ncbi.nlm.nih.gov/books/NBK448105/.

